# Comparing ion torrent with pyrosequencing and Sanger sequencing for the detection of *TP53* mutations in colorectal cancer

**DOI:** 10.1186/1878-5085-5-S1-A32

**Published:** 2014-02-11

**Authors:** Brendan Doyle, Clarence C Lee, Timothy T Harkins, Rosella Petraroli, Paul Smyth, Kieran Sheahan, John J O’Leary, Orla Sheils

**Affiliations:** 1 Department of Histopathology, Trinity College Dublin, Ireland; 2 Life Technologies, Foster City, CA, USA; 3 Department of Histopathology, St. Vincent’s University Hospital, Dublin, Ireland

## Scientific objectives

The detection of mutations in tumour tissue is increasingly important to pathologists due to the number of targeted, personalised therapies that are only effective in tumours displaying specific mutations. At present, much of the testing to detect these mutations is performed using individual assays. This has proven to be an effective method as the number of ‘drugable’ mutations is currently relatively low. However, the number of targeted therapies (and therefore targets to be assessed) is expected to rise significantly. This has led to the development of assays capable of assessing mutations across multiple gene panels. Here we assess one new technology (Ion Torrent’s PGM), comparing the results to those obtained with pyrosequencing and Sanger sequencing, both established technologies used in molecular pathology laboratories.

## Technological approach

DNA extracted from 8 cell lines with known *TP53* mutations (CCRF-CEM, SW 837, NCI-H23, U251, MDAMD231, SKBR-3, CALU6, C33a) was sequenced using Ion Torrent (Ion PGM sequencer, Life Technologies), Pyrosequencing (PyroMark Q24, Qiagen) and Sanger sequencing technology.

## Results

The Ion Torrent results matched those achieved using pyrosequencing and Sanger sequencing in all 8 cases. The results also matched the known mutations for these cell lines in the TP53UMD mutation database.

## Conclusion and recommendations

Results achieved with the Ion Torrent PGM were comparable to those seen using pyrosequencing and Sanger sequencing. As the number and complexity of tests required to inform clinicians increases technologies such as this, with high throughput and scope for expansion may prove beneficial to diagnostic molecular pathology laboratories.

